# An Interesting Operation for Ovarian Cyst

**Published:** 1891-06

**Authors:** Jno. R. Bradfield, Fred’k. M. Reeves

**Affiliations:** Alvord, Texas; Alvord, Texas


### AN INTERESTING OPERATION FOR OVARIAN CYST.

By JNO. R. BRADFIELD, M. D., AND FRED'K. M. REEVES, M. D.,
ALVORD, TEXAS.

The patient, Mrs. E. C., æt. 42, came to us on the 10th ult., suffering with amenorrhœa, which was partially relieved by the usual treatment, prior to a thorough examination to establish the existence of an ovarian *cyst*, which patient said had been diagnosed as such four years previously, and operative measures for removal were refused, owing to the size of tumor and condition of patient at that time. We were called later and found her in very pronounced hystero-epileptic convulsions, which were finally overcome in the course of six or eight hours, leaving her wellnigh exhausted. On making a thorough examination, we found, *per vaginam*, a hard immovable cyst lying in the ovarian region—characteristic in fact.

The aspirator showed a small quantity of serous fluid and disorganized tissue.

We were satisfied that the only alternatives were an operation or death, because the tumor was degenerating and no doubt would sooner or later break down and produce death by peritonitis or some other complication. We prepared patient for operation on the 20th ult. After having cleansed the parts thoroughly with 1 to 40 carb. sol., we made an incision sufficiently extensive to allow us room for removal in the linea alba, taking considerable care to leave wound with smooth edges, catching up the bleeding points—leaving nothing to enter the cavity. The tumor, or cyst rather, was laid bare, an assistant clasping edges of wounds *down* against the surface of the cyst, leaving only sufficient space for making an incision; opened the cyst with one stroke of the scalpel, allowed half the contents, the whole being perhaps a quart and a half, at least, to escape without contaminating the external wound, then passing a strong cat-gut ligature around the thin walls, had it as secure as when intact and much more space to break up adhesions and secure arteries. We severed the fallopian tube three or four lines from the uterus.

After removal we packed the cavity with antiseptic gauze until perfectly dry, there having been very little hemorrhage and no contact with contents of cyst. We then caught up the edges of the peritoneum in the lips of the wound, securing same with large cat-gut sutures at every three or four lines and intermediate silk sutures bringing edges smoothly together. We left drainage tube in lower part. We used a very large quantity of iodoform over the surface generally. Then the dressing was completed with absorbent cotton held by eight-inch bandages, followed, in anticipation of shock, with morphine and verat. viride, and watched the general condition of patient for forty-eight hours, the one or the other attending. The temperature ranged below 101° ; the bowels were "moved" by enemata. The patient bids fair to recover at this time.

The main feature of the case was not the size of the tumor, but its slow progress.

